# Deep Learning Detection of Early Retinal Peripheral Degeneration From Ultra-Widefield Fundus Photographs of Asymptomatic Young Adult (17–19 Years) Candidates to Airforce Cadets

**DOI:** 10.1167/tvst.13.2.1

**Published:** 2024-02-01

**Authors:** Tengyun Wu, Lie Ju, Xuefei Fu, Bin Wang, Zongyuan Ge, Yong Liu

**Affiliations:** 1 Air Force Medical Center of Chinese PLA, Beijing, China; 2 Beijing Airdoc Technology Co. Ltd., Beijing, China; 3 Faculty of engineering, Monash University, Clayton, Australia

**Keywords:** artificial intelligence, ultra-widefield fundus, retina, Air Force cadets’ medical selection

## Abstract

**Purpose:**

Artificial intelligence (AI)–assisted ultra-widefield (UWF) fundus photographic interpretation is beneficial to improve the screening of fundus abnormalities. Therefore we constructed an AI machine-learning approach and performed preliminary training and validation.

**Methods:**

We proposed a two-stage deep learning-based framework to detect early retinal peripheral degeneration using UWF images from the Chinese Air Force cadets’ medical selection between February 2016 and June 2022. We developed a detection model for the localization of optic disc and macula, which are used to find the peripheral areas. Then we developed six classification models for the screening of various retinal cases. We also compared our proposed framework with two baseline models reported in the literature. The performance of the screening models was evaluated by area under the receiver operating curve (AUC) with 95% confidence interval.

**Results:**

A total of 3911 UWF fundus images were used to develop the deep learning model. The external validation included 760 UWF fundus images. The results of comparison study revealed that our proposed framework achieved competitive performance compared to existing baselines while also demonstrating significantly faster inference time. The developed classification models achieved an average AUC of 0.879 on six different retinal cases in the external validation dataset.

**Conclusions:**

Our two-stage deep learning–based framework improved the machine learning efficiency of the AI model for fundus images with high resolution and many interference factors by maximizing the retention of valid information and compressing the image file size.

**Translational Relevance:**

This machine learning model may become a new paradigm for developing UWF fundus photography AI-assisted diagnosis.

## Introduction

The advent of ultra-widefield fundus imaging (UWF) has made it possible to observe almost the entire fundus through a nonmydriatic pupil with a 200° view,^1^ including the posterior pole and peripheral regions.^2^ With eye position guidance, we can observe almost all retinal conditions.

The UWF fundus photography technology (Dytona; Optomap, Dunfermline, UK) was applied in the medical selection of Air Force cadets of the Chinese People's Liberation Army for several years. According to our experience, the application of UWF laser fundus photography improves the efficiency of fundus examination by more than 30% compared with the traditional examination mode in Chinese Air Force cadets’ medical selection. Thus UWF photography has become a necessary fundus examination tool for the medical selection of Chinese Air Force cadets. According to our previous study, peripheral retinal degeneration (including snail track degeneration, lattice degeneration, microcystic degeneration), white without pressure, and vitreoretinal tuft are the most common peripheral retinal diseases observed during medical selection of Chinese Air Force cadets.^3^ Studies have shown that these signs do not carry a high risk of clinical events such as retinal detachment or vitreous hemorrhage, and regular examinations are generally recommended in clinical work,^4^^,^^5^ except for lattice degeneration, which is directly related to retinal detachment in 20%.^6^ However, the medical risk of these abnormal signs may increase when piloting an airplane, especially in the situation of high acceleration.^7^ Meanwhile, these peripheral retinopathies are mostly progressive. Follow-up and timely intervention are necessary.

UWF imaging can help identify diabetic retinopathy, retinal detachment, macular holes, pathological myopia,^8^^–^^11^ and so on. However, the interpretation of UWF images requires professional retinal skills, which limits the wide application in grassroots units. Therefore, an automated intelligent diagnosis system based on deep learning has been developed to improve the accuracy of image diagnosis. Currently, research on deep learning systems using UWF images has mostly focused on the detection of glaucomatous optic neuropathy, retinal exudates, and drusen.^12^^,^^13^ However, these retinal disease detection models have limited application in the medical selection of Air Force cadets and in recognition of early peripheral retinopathy. To date, no automated intelligent systems have been reported to detect early peripheral retinal degeneration or physiological changes. In addition, an effective model of AI assistant image diagnosis requires a huge sample for deep machine learning. Traditional deep learning-based methods take the resized image (e.g., 224 × 224) as input. However, UWF fundus images are high-resolution with about 2000 to 3000 pixels, and resizing these images may result in a loss of important details, such as some early lesions, which are always small and can be easily ignored. In addition, to increase the sensitivity of detecting peripheral lesions, four directions of eye position guidance are needed when taking fundus images.^14^ To solve these problems, in this study, we developed a deep learning system for automated detection of early peripheral retinal degeneration using UWF images. The proposed system enhances the accuracy of lesion detection in peripheral retinal areas, which greatly improves the identification rate of peripheral lesions.

## Methods

### Label Setting

**Figure 1.**
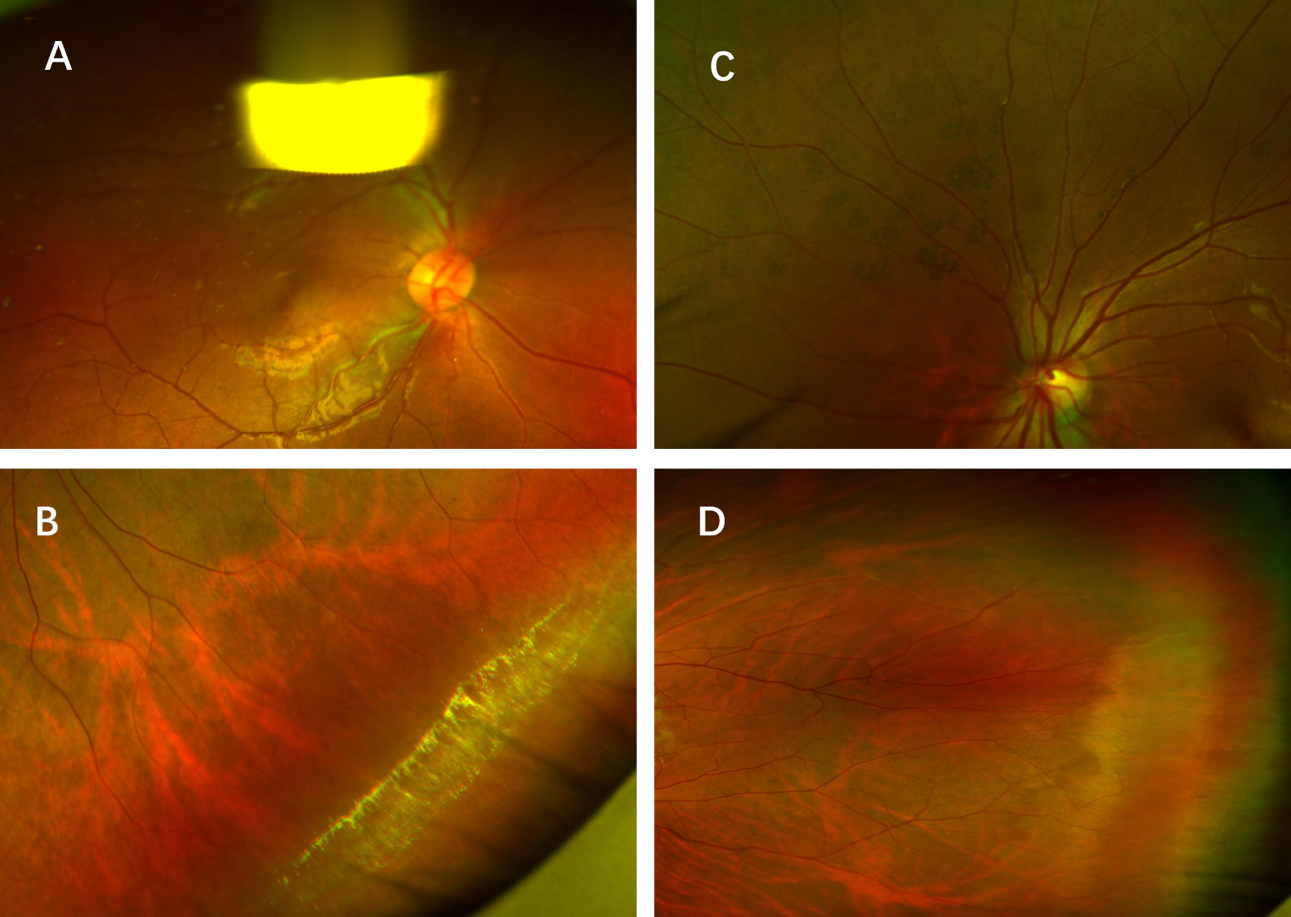
**Schematic diagram of label setting.** (**A**) A typical example for label “facula”, which was defined as a block highlight area. (**B**) A typical example for label “degeneration”. (**C**) A typical example for label “hyperpigmentation”. (**D**) A typical example for label “WWOP”.

In total six labels were chosen in our study. “Normal/abnormal” was defined as whether there was an abnormal sign in the fundus image; “facula” was defined as a block highlight area, as is shown in Figure 1A; “degeneration” was defined as various types of peripheral retinal degeneration, such lattice degeneration or snail trace-like degeneration (shown in Fig. 1B); “hyperpigmentation” and “hypopigmentation” were defined as choroidal pigment epithelium or retinal pigment epithelium hyperplasia or atrophy (shown in Fig. 1C); and label “WWOP” was defined as white without pressure, which is quite common in clinical practice (shown in Fig. 1D).

### Datasets Collection

All fundus images were collected from the medical selection of the Chinese Air Force cadets’ medical selection between February 2016 and June 2022. Students’ fundus images were taken under nonmydriasis status by the coauthor Tengyun WU, and use the eye position guidance function of UWF photography scanning system (Daytona; Optos, Dunfermline, UK) to collect the upper, lower, nasal, and temporal fundus images, respectively.

All these images were reviewed by four retinal specialists independently. To ensure the accuracy of target lesions, the same images were sent anonymously and independently to two retinal specialists who have extensive medical selection experience. When two retinal specialists reached a consensus regarding identification outcome, the images were used for subsequent model training. Inconsistency resulted in a discussion among retinal specialists, and images that did not have consistent results were discarded. If the image in the fundus photograph was significantly deformed or the image of eyelid obscured the area behind the vortex vein, then this photograph was excluded from our study. Because interference, such as occlusion and reflection, needs to be learned during AI machine training, the occurrence of reflection or occlusion on fundus photographs that do not interfere with the recognition of fundus signs is not an exclusion standard. As is shown in the pipeline, a total of 4023 images were used for AI machine learning, and 760 images were used to validate the performance of the diagnostic model.

### Development of a Deep-Learning System

#### Image Preprocessing

To demonstrate the effectiveness of our proposed methods, we investigated three preprocessing techniques:

##### Resizing Method

For all raw images (with resolution about 2000 to 3000 pixels), which were annotated as positive or negative, they were resized into a size of 512 × 512 pixels. Our model takes a mini-batch of the resized images as inputs for training. It is obvious that the “resizing method” does not require any pixel-level annotation like bounding box for the location of lesions.

##### Patch-Based Method

For all raw images, they were first resized to 3200 × 3200 pixels. Then, a sliding window with the size of 512 × 512 pixels was used to extract patches from the resized raw images with the stride of 256. To obtain the patch-level annotation for each extracted patch, a threshold *t* was set. Specifically, we calculated the areas of lesions occupied in the patch. Those patches whose areas of lesions exceed *t* were annotated as “positive”; those that did not were annotated as “negative.” Twenty positive patches were selected for each lesion blob. For each lesion blob, five different positions of patch center were randomly selected around the center of the lesion blob with four different sizes (512 × 512, 640 × 640, 720 × 720, 896 × 896). Finally, those patches are resized into 512 × 512. The model takes a mini-batch of the patches as inputs for training. In the inference stage, we took the max value of all patches extracted from one image as the positive score. Compared with the “resizing method,” the “patch-based method” requires pixel-level annotation and more inference time.

##### Edge-Sensitive Method

In this study, we care about those lesions existing around the edge area of the UWF fundus image. First, we trained a model, which was leveraged to detect out the locations of optic disc and macula. Then, we calculated the distances of the optic disc to the edge of four directions (up, bottom, left, and right). The edge of the direction with the largest distance could be regarded as the “real” edge of the fundus. Given the original image with size (0,0)∼(w,h) (from top-left to bottom-right), the locations of optic disc $\left(x_{1},y_{1}\right)$ and macula $\left(x_{2},y_{2}\right)$, the distance between optic disc and macula can be calculated as $r=\sqrt{{\left(x_{1}-x_{2}\right)}^{2}+{\left(y_{1}-y_{2}\right)}^{2}}$ and the center of two objects can be formulated as $\left(x_{3},y_{3}\right)=\left(\frac{x_{1}+x_{2}}{2},\frac{y_{1}+y_{2}}{2}\right)$. We first calculate the distances of the optic disc to its left edge and right edge with w_1_ = x_1_ − 0 and w_2_ = w − x_1_. Then we calculate the distances of the optic disc to its top edge and bottom edge with h_1_ = y_1_ − 0 and h_2_ = h − y_1_. We have the following ties to obtain the edge area:

a. If $h_{1}<\frac{h}{3}$, we crop the bottom edge (0, y_3_ + 2*r) ∼ (w, h) as the edge area.
b. If $h_{1}>\frac{2*h}{3}$, we crop the top edge (0, 0) ∼ (w, y_3_ − 2*r) as the edge area.
c. If $\frac{h}{3}<h_{1}<\frac{2*h}{3}$ and $w_{1}<\frac{w}{3}$, we crop the right edge (x_3_ + 2*r, 0) ∼ (w, h) as the edge area.
d. If $\frac{h}{3}<h_{1}<\frac{2*h}{3}$ and $w_{1}>\frac{2*w}{3}$, we crop the left edge (0, 0) ∼ (x_3_ − 2*r, h) as the edge area.
e. If $\frac{h}{3}<h_{1}<\frac{2*h}{3}$ and $\frac{w}{3}<w_{1}<\frac{2*w}{3}$, we compare the value of w_1_ and w_2_. If w_1_ > w_2_, we crop the left edge and vice versa.

The cropped the edge area was then resized into the desired resolutions and taken as the input of the model. Our proposed “edge-sensitive method” shows that the ability and sensitivity of early detection of those lesions occurs in the edge of the fundus such as retinal peripheral degeneration.

#### Model Building

Our proposed edge-sensitive preprocessing method necessitates the precise localization of the optic disc and macula. To achieve this, we use the YOLOv3^18^ detector, a state-of-the-art deep learning architecture designed for object detection tasks. YOLOv3 is known for its real-time object detection capabilities, and it uses a deep convolutional neural network that divides the input image into a grid and simultaneously predicts bounding boxes and class probabilities for objects within each grid cell. This architecture is highly efficient and has demonstrated remarkable accuracy in object detection.

For the subsequent classification task, where we diagnose multiple retinal lesions, we use the ResNet-50^15^ model. ResNet-50 is a variant of the Residual Network (ResNet) architecture, which is celebrated for its ability to train very deep neural networks effectively. ResNet-50 comprises 50 layers and incorporates residual connections that enable the smooth flow of gradients during training. It has been widely adopted in computer vision tasks and is particularly well suited for capturing intricate features within images, making it an excellent choice for our classification model.

#### Implementation Details

All input images were resized to 512 × 512 pixels before their use in the detection and classification network. The pixel values of each fundus images were normalized from (0, 255) to (0, 1) before the model training. To obtain more samples for training, we apply some data augmentation techniques such as horizontal and vertical flips. The Adam optimizer was used for backpropagation. For the yolov3 detector, the learning rate was set as 1 × 10^−4^ with weight decay of 5 × 10^−4^. For the classification model, the learning rate was originally set as 1 × 10^−3^ and the division by 10 on epoch 10 and epoch 20. The total 50 epochs were trained. All the experiments were performed using Ubuntu version 18.04.4 LTS 64-bit with GPU 3090 and 24 GB memory. The implementation of deep neural networks was based on PyTorch platform version1.6.0.

#### Internal Validation

Images with the cared lesions were randomly assigned in a 4:1 split to training sets and internal validation sets. All diagnosis models were trained on training images with the specific lesions, and the internal validation sets were used to determine which checkpoint we should select during the training phase for the external validation. Statistical performance for the classifier was measured by calculating the area under the receiver operating characteristic curve (AUROC).

#### External Validation

To test the performance of the diagnostic model in a real-world, 760 images were independently collected to form the external validation sets and were not included in the training or internal validation sets. Statistical performance for the classifier was measured by calculating AUROC, sensitivity, specificity, precision, and F1 score. The threshold for the prediction probability from the binary classification model is selected using the Youden index.

## Results

### Automatic Preprocessing Versus Ophthalmologists’ Manipulation

To evaluate the effectiveness of our proposed edge-sensitive preprocessing method, we recruited two general ophthalmologists who had more than two years of experience in UWF fundus image examination. For randomly-selected 100 UWF fundus images, they were asked to find the peripheral area of each image and manually crop the desired areas. Then we compared the model outputs and results from the ophthalmologists. Among 100 images, only two images were wrongly cropped. We reviewed these two images, and it was found that the eyes in these two images are both in the primary position, which was not of concern or suitable in our study.

### On Evaluation of Detection of Optic Disc and Macula

**Figure 2.**
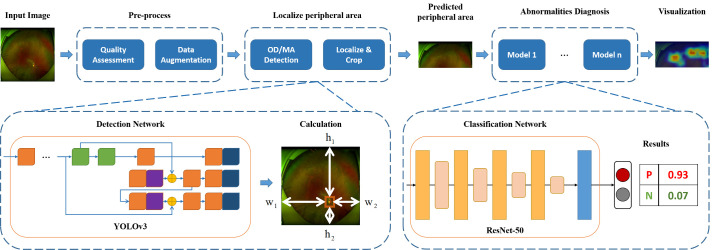
**Study framework of the proposed methods for the detection.** OD, optic disc; MA, macular.

To obtain the peripheral area for the following process, the accuracy of the detection of the optic disc and macula should be promised. In Figure 2 and Table 1, we report the performance of the detection of optic disc and Macula using different widely-used object detection framework (e.g., SSD,^16^ Faster-RCNN,^17^ YOLOv3).^18^ The detection accuracy is calculated as the ratio of number of successful detected object and total number of objects. It was found that YOLOv3 achieved the highest accuracy and other frameworks also achieved competitive performance. In 116 and 115 images out of 117 validation images, optic disc and Macula were successfully detected, respectively.

**Table 1.** Detection Accuracy of OD and Macula Using Different Object Detection Network

Method	OD	Macula
SSD	99.15%	93.16%
Faster-RCNN	98.29%	97.43%
YOLOv3	99.15%	98.29%

OD, optic disc; SSD, single shot multibox detector.

### Prospective Validation Results in Screening Setting

**Figure 3.**
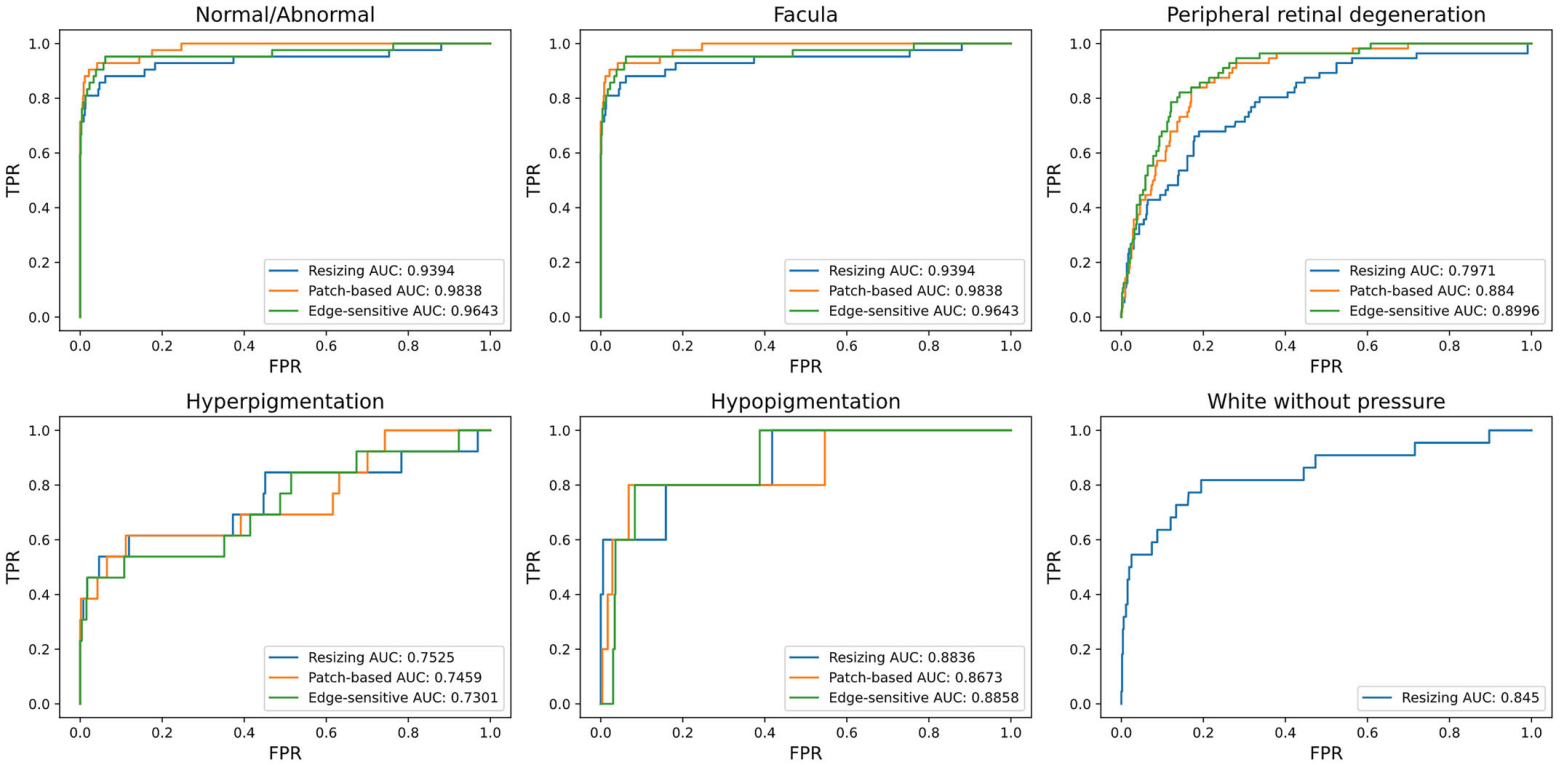
The comparisons of Resizing method's, Path-based method's and Edge-sensitive method's (our proposed method's) AUROC curves with 6 cases classification on the external validation dataset.

The comparisons of the model performance between our developed framework and other comparison methods in six cases are shown in Figure 3 and Table 2. For the original resize method, the AUROC score, sensitivity, specificity, and F1 score for identifying Normal/Abnormal reached 0.9394 (0.8889–0.9898), 0.8571 (0.7846–0.9297), 0.9387 (0.8879–0.9894), and 0.5950 (0.5022–0.6879), respectively. For other observed cases achieved the AUROC (95% confidence interval [CI]) of facula 0.9525 (0.9221–0.9828), peripheral retinal degeneration 0.7971 (0.7255–0.8687), hyperpigmentation 0.7525 (0.5978–0.9073), and hypopigmentation 0.8836 (0.6906–1.0000).

**Figure 4.**
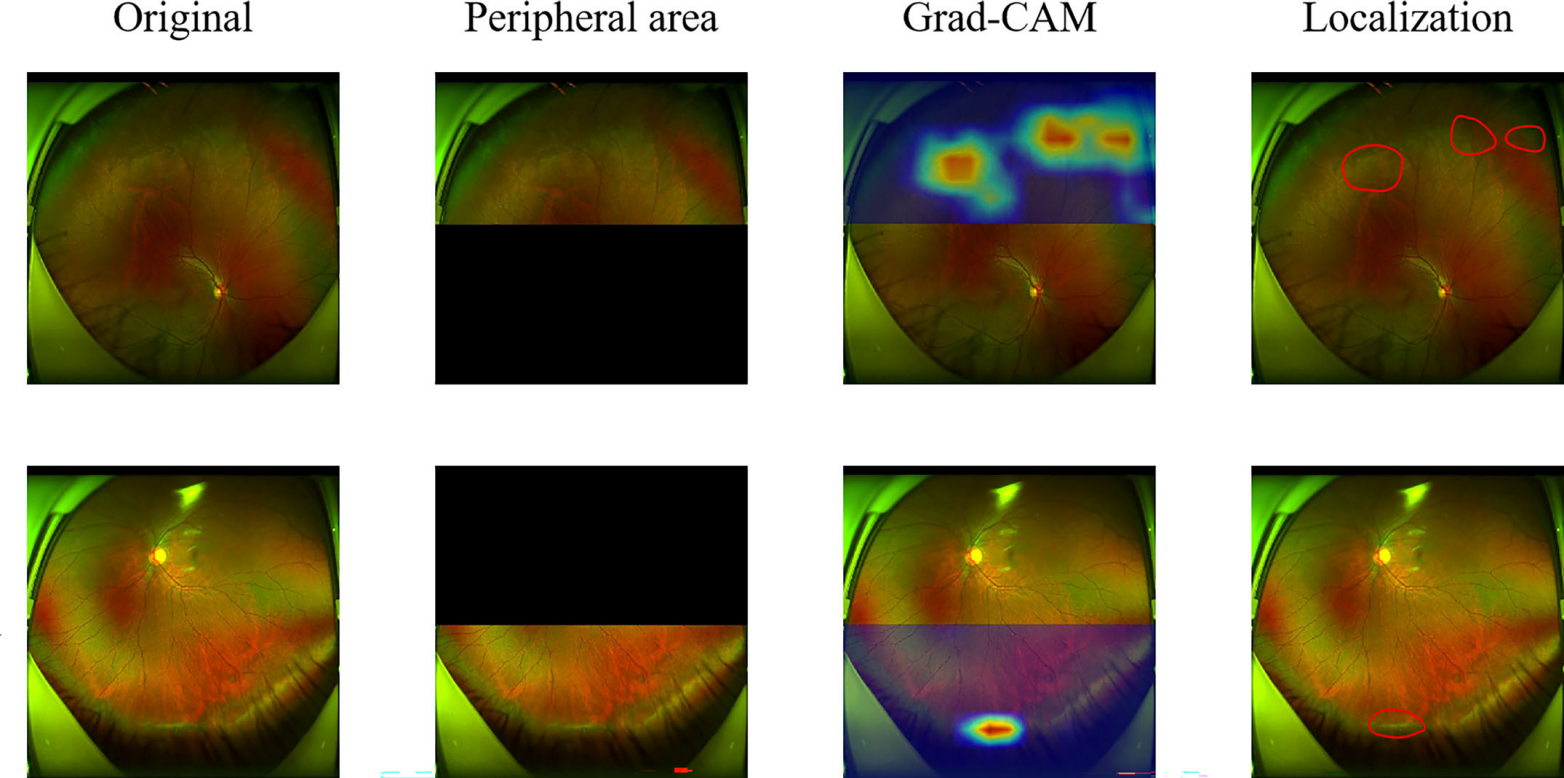
**The Grad-CAM visualization results and corresponding ground truth for the detection of retinal peripheral lesions.** In this study, Grad-CAM is applied for the visualization analysis.

**Table 2.** AUC, Sensitivity, Specificity, Precision, and F1 Performance of Different AI Model

	Resizing Method	Patch-Based Method	Edge-Sensitive Method
Normal/Abnormal			
AUC	0.9394 (0.8889–0.9898)	0.9838 (0.9569–1.0000)	0.9643 (0.9248–1.0000)
Sensitivity	0.8571 (0.7846–0.9297)	0.9048 (0.8432–0.9663)	0.9286 (0.8743–0.9829)
Specificity	0.9387 (0.8879–0.9894)	0.9586 (0.9164–1.0000)	0.9387 (0.8879–0.9894)
Precision	0.4557 (0.3686–0.5428)	0.5672 (0.4747–0.6596)	0.4756 (0.3871–0.5641)
F1	0.5950 (0.5022–0.6879)	0.6972 (0.6066–0.7879)	0.6290 (0.5363–0.7218)
Facula			
AUC	0.9525 (0.9221–0.9828)	0.9534 (0.9233–0.9834)	0.9537 (0.9238–0.9837)
Sensitivity	0.9149 (0.8753–0.9545)	0.9468 (0.9149–0.9788)	0.9149 (0.8753–0.9545)
Specificity	0.8977 (0.8548–0.9406)	0.8864 (0.8415–0.9312)	0.8996 (0.8571–0.9422)
Precision	0.6143 (0.5497–0.6789)	0.5973 (0.5326–0.6620)	0.6187 (0.5542–0.6832)
F1	0.7350 (0.6744–0.7957)	0.7325 (0.6717–0.7933)	0.7382 (0.6778–0.7986)
Degeneration			
AUC	0.7971 (0.7255–0.8687)	0.8840 (0.8259–0.9422)	0.8996 (0.8449–0.9544)
Sensitivity	0.6607 (0.5798–0.7416)	0.8214 (0.7528–0.8900)	0.8036 (0.7327–0.8744)
Specificity	0.8106 (0.7406–0.8806)	0.8295 (0.7621–0.8970)	0.8580 (0.7949–0.9210)
Precision	0.2701 (0.2110–0.3292)	0.3382 (0.2709–0.4056)	0.3750 (0.3039–0.4461)
F1	0.3834 (0.3116–0.4553)	0.4792 (0.4006–0.5578)	0.5114 (0.4313–0.5914)
Hyperpigmentation			
AUC	0.7525 (0.5978–0.9073)	0.7459 (0.5902–0.9016)	0.7301 (0.5722–0.8880)
Sensitivity	0.5385 (0.3762–0.7007)	0.5385 (0.3762–0.7007)	0.3846 (0.2427–0.5265)
Specificity	0.8810 (0.7601–1.0000)	0.8887 (0.7710–1.0000)	0.9827 (0.9328–1.0000)
Precision	0.1014 (0.0483–0.1546)	0.1077 (0.0518–0.1635)	0.3571 (0.2209–0.4934)
F1	0.1707 (0.0894–0.2520)	0.1795 (0.0950–0.2640)	0.3704 (0.2313–0.5094)
Hypopigmentation			
AUC	0.8836 (0.6906–1.0000)	0.8673 (0.6642–1.0000)	0.8858 (0.6944–1.0000)
Sensitivity	0.6000 (0.3359–0.8641)	0.6000 (0.3359–0.8641)	0.6000 (0.3359–0.8641)
Specificity	0.8412 (0.6242–1.0000)	0.9319 (0.7784–1.0000)	0.9168 (0.7491–1.0000)
Precision	0.0345 (0.0029–0.0660)	0.0769 (0.0122–0.1417)	0.0638 (0.0090–0.1186)
F1	0.0652 (0.0093–0.1211)	0.1364 (0.0299–0.2428)	0.1154 (0.0231–0.2077)
WWOP			
AUC	NA	NA	0.8450 (0.7420–0.9481)
Sensitivity	NA	NA	0.7727 (0.6560–0.8895)
Specificity	NA	NA	0.8053 (0.6939–0.9168)
Precision	NA	NA	0.1429 (0.0871–0.1986)
F1	NA	NA	0.2411 (0.1586–0.3237)

For the cropped method, the AUROC score, sensitivity, specificity, and F1 score for identifying Normal/Abnormal reached 0.9838 (0.9569–1.0000), 0.9048 (0.8432–0.9663), 0.9586 (0.9164–1.0000), and 0.6972 (0.6066–0.7879), respectively. For other observed cases achieved the AUROC (95% CI) of facula 0.9534 (0.9233–0.9834), peripheral retinal degeneration 0.8840 (0.8259–0.9422), hyperpigmentation 0.7459 (0.5902–0.9016), and hypopigmentation 0.8673 (0.6642–1.0000).

For our proposed edge-sensitive method, the AUROC score, sensitivity, specificity, and F1 score for identifying Normal/Abnormal reached 0.9643 (0.9248–1.0000), 0.9286 (0.8743–0.9829), 0.9387 (0.8879–0.9894), and 0.6290 (0.5363–0.7218), respectively. For other observed cases achieved the AUROC (95% CI) of facula 0.9537 (0.9238–0.9837), peripheral retinal degeneration 0.8996 (0.8449–0.9544), hyperpigmentation 0.7301 (0.5722–0.8880), and hypopigmentation 0.8858 (0.6944–1.0000). For white without pressure, two comparison methods did not achieve satisfactory results and our proposed method achieved the AUROC (95% CI) of 0.8450 (0.7420–0.9481).

### Time-Consuming Analysis

Because we applied different pre-processing techniques, the efficiency and time-cost should be considered. For the original resizing method, the model took the whole image as the input during the training and inference phase. For the patch-based method, 20 positive patches were cropped during the training phase. During the inference phase, a sliding window was leveraged to crop an average of 16 to 20 patches as the inputs to the model. Hence, the model took 20 and 16 to 20 times longer than the original resizing method during the training and testing phases, respectively. For our proposed edge-sensitive method, the original image was fed into two deep neural networks and took about only two times longer than the original resizing method both in the training and inference time. A detailed comparison is shown in Table 3.

**Table 3.** Comparison of Training and Testing Time Consumption for Three Preprocessing Techniques

Method	Training	Test	Precision
Original	1x	1x	Low
Cropped	16x–20x	16x	High
Edge-sensitive	1x	1x	High

### Heatmap Visualization

In this study, Grad-CAM^19^ is applied for the visualization analysis. The results are shown in Figure 4. The highlighted regions denote the pixels that contribute most to the diagnostic results. The regions of interest also match with lesions that ophthalmologists would pay attention to when making the diagnosis.

## Discussion

The application of UWF photography helps ophthalmologists to have a better observation of the fundus, because it can collect the information of about 200° of the fundus and present it on a photograph with a resolution of 4 µm in less than one second. It has become an essential part of the medical selection of Chinese Airforce cadets. In our experience, the use of UWF photography has significantly improved screening efficiency, especially when we need to observe retinopathy in the far periphery.

AI-assisted diagnosis is rapidly developing in clinical disease diagnosis and management. It has made promising progress in the screening and follow-up of fundus diseases such as age-related macular degeneration, diabetic retinopathy, and glaucoma.^20^^–^^23^ However, the technology of AI-assisted early peripheral retinopathy diagnosis using UWF imaging as a detection object in healthy populations is still immature. In addition, AI-assisted early peripheral retinopathy screening for Air Force flying cadets has not yet been addressed.

Compared to traditional color fundus photography, UWF imaging has a much larger document size and resolution ratio, as well as more interference information. Therefore it is difficult to use the whole image for AI machine learning. If we reduce the resolution of the images, a lot of information will be lost, which will significantly reduce the accuracy of the AI model in identifying peripheral retinal lesions. As reported in a previous study,^11^ some researchers cut the whole fundus image into several small blocks to reduce the input size of each image before machine learning. However, this preprocessing method may increase the time required for machine learning. It is necessary to develop an image preprocessing method that can not only solve the problem of large image resolutions but also maximize the retention of useful information.

In clinical practice, eye position guidance is the key factor that can improve the detection rate of peripheral retinopathy.^14^ Thus four-direction eye position guidance has become a routine examination in the medical selection of Chinese Air Force cadets and has becoming increasingly important in clinical work. The AI model needs to have deep learning of the images acquired after eye position guidance so that it can better handle the images acquired in clinical practice.

Taking these factors into consideration, in this study we developed a new image preprocessing scheme. As is shown in Figure 2, we leverage an object detection network to detect the location of optic disc and macula; then the edge of UWF could be located following the ties accordingly. We first evaluate the effectiveness of our proposed edge-sensitive preprocessing method. Then, all images those used in the machine learning were preprocessed using this method.

As shown in Table 1, our model performs equally well in identifying peripheral retinopathy compared with that reported in the previous literature. Differently, as we use edge-sensitive preprocessing logic, the preprocessing process can ensure the retention of peripheral lesion information. In addition, as is shown in Table 2, the time consumed in both the training and testing phases is significantly reduced. Our research provides a new image preprocessing and training scheme for AI machine learning with high-resolution images that can effectively improve learning efficiency.

It is noteworthy that our model showed clear advantages in the identification of WWOP. Although it is not difficult to diagnose WWOP clinically, its features are not very obvious in fundus photography. In this case, both the resizing method and the patch-based method will cause a lot of information loss, and our “edge sensitive” method demonstrated a significant advantage in maximizing the retention of valid information. Therefore we speculate that our model may have more advantages in identifying lesions with large area and insignificant imaging features, such as WWOP, dark without pressure (DWOP), and peripheral retinal superficial detachment.

In general, the medical selection of Chinese Air Force cadets is the basis for the construction of air force combat power. Therefore, how to screen quickly and accurately, to reduce misdiagnosis and omission, and to achieve scientific and accurate medical selection is important work related to ensuring future air force pilot safety. Although the clinical significance of peripheral retinal degeneration, WWOP, an more is limited, it still has potential risk in special environments such as flight operations and requires careful examination and careful handling of the fundus. The model in this study uses UWF photography to assist in the diagnosis of early peripheral retinopathy, greatly increasing the efficiency of screening with high accuracy; at the same time, such systematic tools can reduce the workload of the specialists involved and allow objective and efficient screening of patients.

Also, our study provides a viable option for AI machine learning to recognize peripheral fundus abnormalities, which is expected to improve the application of AI-assisted diagnosis in ophthalmology clinical diagnosis in the future.

To the best of our knowledge, this is the first report to establish a deep learning system to detect early peripheral retinal degeneration, WWOP, and pigmentary changes in UWF images with an accuracy level. In addition, this study has several limitations. First, in this study, we focused on the detection of peripheral retinopathy, so our model has poor detection efficiency of pathological changes of the fundus posterior pole. In addition, all the images used for machine learning were selected from teenagers who participated in the medical selection of Air Force cadets. Because our AI-based diagnostic model may not have high detection efficiency for the fundus images of clinical patients, deep learning of clinical cases is needed in the future.

## Supplementary Material

Supplement 1
