# Supplementary figures and images for: Deep Learning Detection of Early Retinal Peripheral Degeneration From Ultra-Widefield Fundus Photographs of Asymptomatic Young Adult (17–19 Years) Candidates to Airforce Cadets
Source: Transl Vis Sci Technol. 2024 Feb 1;13(2):1. doi: 10.1167/tvst.13.2.1 (PMC10851781; doi:10.1167/tvst.13.2.1)

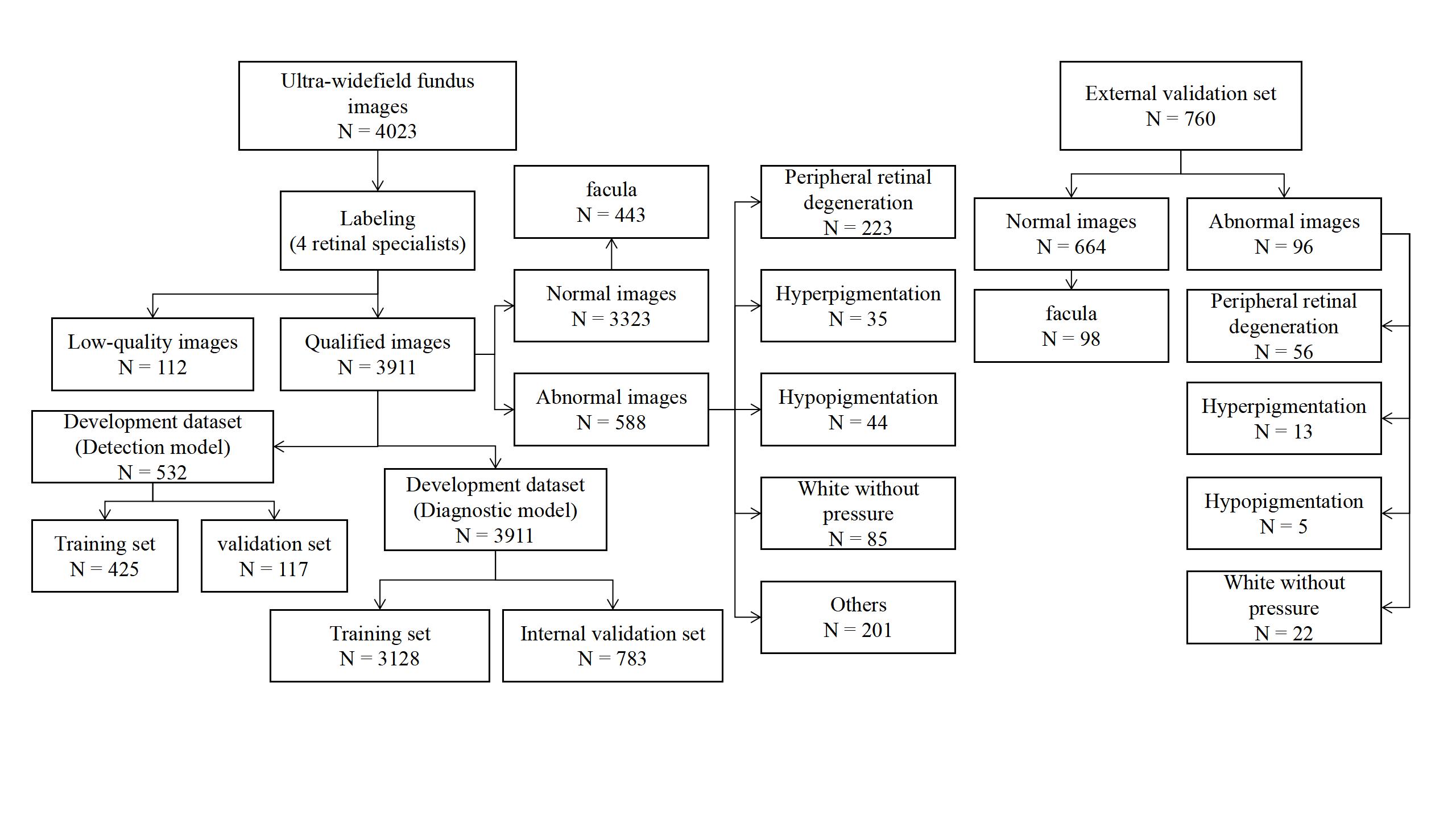

Supplement: Supplement 1 [file tvst-13-2-1_s001.jpg]
